# Aiming for a bull’s-eye: Targeting antifungals to fungi with dectin-decorated liposomes

**DOI:** 10.1371/journal.ppat.1009699

**Published:** 2021-07-22

**Authors:** Richard B. Meagher, Zachary A. Lewis, Suresh Ambati, Xiaorong Lin

**Affiliations:** 1 Department of Genetics, University of Georgia, Athens, Georgia, United States of America; 2 Department of Microbiology, University of Georgia, Athens, Georgia, United States of America; University of Maryland, Baltimore, UNITED STATES

## Abstract

Globally, there are several million individuals with life-threatening invasive fungal diseases such as candidiasis, aspergillosis, cryptococcosis, *Pneumocystis* pneumonia (PCP), and mucormycosis. The mortality rate for these diseases generally exceeds 40%. Annual medical costs to treat these invasive fungal diseases in the United States exceed several billion dollars. In addition to AIDS patients, the risks of invasive mycoses are increasingly found in immune-impaired individuals or in immunosuppressed patients following stem cell or organ transplant or implantation of medical devices. Current antifungal drug therapies are not meeting the challenge, because (1) at safe doses, they do not provide sufficient fungal clearance to prevent reemergence of infection; (2) most become toxic with extended use; (3) drug-resistant fungal isolates are emerging; and (4) only one new class of antifungal drugs has been approved for clinical use in the last 2 decades. DectiSomes represent a novel design of drug delivery to drastically increase drug efficacy. Antifungals packaged in liposomes are targeted specifically to where the pathogen is, through binding to the fungal cell walls or exopolysaccharide matrices using the carbohydrate recognition domains of pathogen receptors. Relative to untargeted liposomal drug, DectiSomes show order of magnitude increases in the binding to and killing of *Candida albicans*, *Cryptococcus neoformans*, and *Aspergillus fumigatus* in vitro and similarly improved efficacy in mouse models of pulmonary aspergillosis. DectiSomes have the potential to usher in a new antifungal drug treatment paradigm.

## Why is there a need for improved efficacy of antifungal drugs?

**Fig 1:**
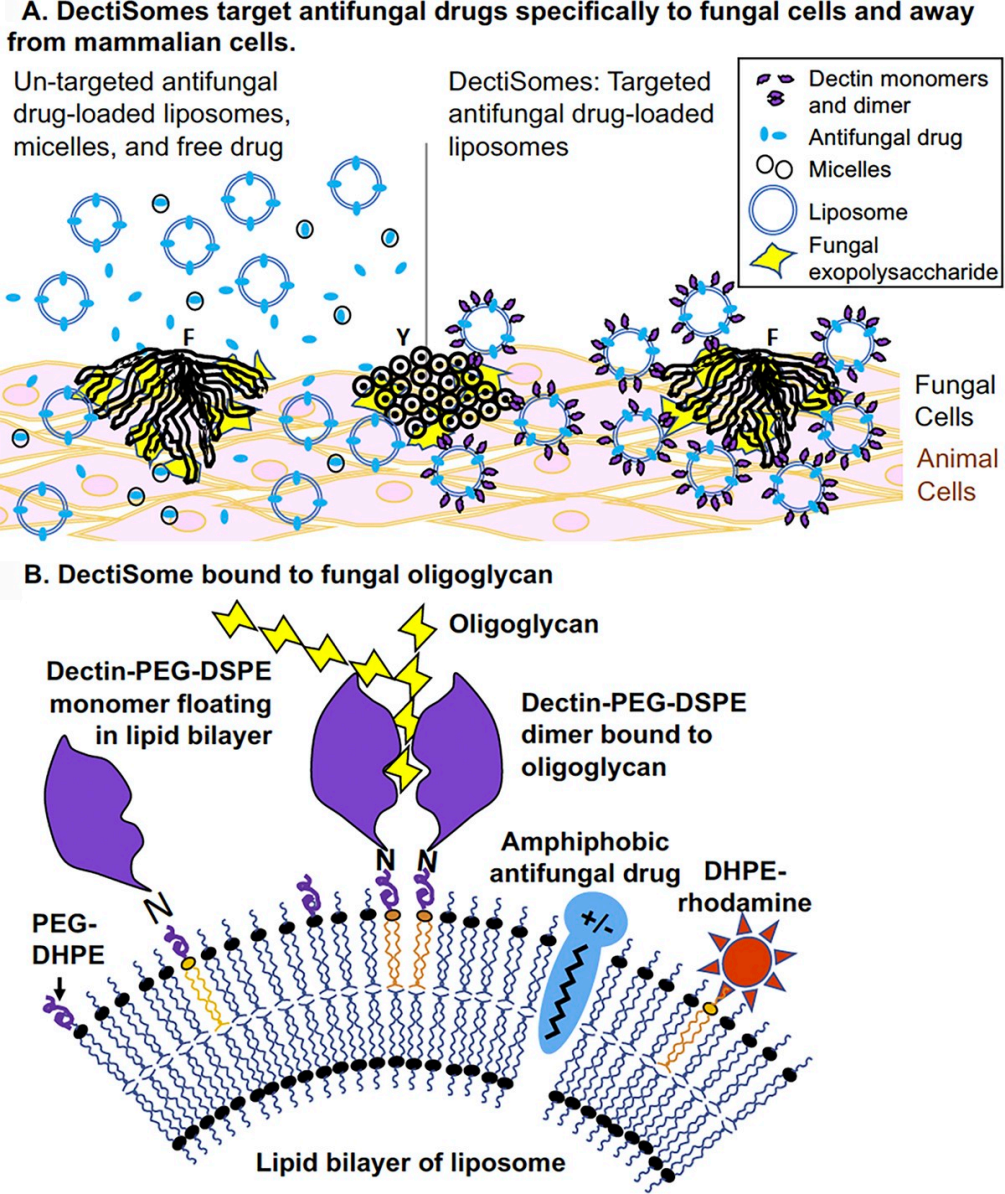
DectiSomes are designed to target antifungal drugs specifically to fungal cells. **(A)** Coating antifungal drug-loaded liposomes with glycan-binding proteins concentrates drugs on fungal cells. Current antifungal drugs are untargeted (left side). Having no selective affinity for fungal cells over host cells demands higher doses of antifungal drugs to inhibit or kill fungal cells. DectiSomes (right side) are drug-loaded liposomes coated with the glycan recognition domains of glycan-binding host receptors such as dectins. They bind specifically to fungal cell walls and their extracellular matrices within biofilms, which reduce the effective dose needed to clear fungal infections and host toxicity. Infection sites composed of fungal cells with Y or F morphologies are shown. (**B)** Structure of a DectiSome. One iteration of a DectiSome is an antifungal drug-loaded liposome coated with the glycan recognition domain of a Dectin. We coupled its glycan-binding domain (purple globular structure) to a lipid carrier to make DEC-PEG-DSPE, which is intercalated into the liposomal membrane via the DSPE moiety. Dectin monomers floating in the liposome membrane form dimers as they bind to fungal oligoglycans (yellow sugar moieties). Rhodamine-B-DHPE is intercalated via its DHPE moiety to allow fluorescent monitoring of liposome binding to fungal cells. Amphiphobic antifungal drugs (blue ovoid structure) are positioned within the lipid bilayer of liposomes. Each approximately 100-nm diameter liposome contains approximately 1,500 Dectin monomers, thousands of antifungal drug molecules, and 3,000 rhodamine molecules. DEC, dectin; DHPE, dihexadecanoyl-glycero-phospho-ethanolamine; DSPE, distearoyl-glycero-phospho-ethanolamine; F, filamentous; PEG, poly(ethylene glycol); Y, yeast.

Several fungal pathogens account for the vast majority of invasive mycoses: There are globally approximately 750,000 cases of candidiasis, 500,000 cases of *Pneumocystis* pneumonia (PCP), 3,000,000 cases of pulmonary aspergillosis, 220,000 cases of cryptococcosis, and 900,000 cases of mucormycosis each year [1,2]. The annual medical costs in the US in 2017 from invasive *Aspergillus*, *Candida*, *Pneumocystis*, *Cryptococcus*, and *Mucor* species-related infections were 1.5 billion, 3 billion, 600,000 million, 200 million, and 120 million US dollars, respectively [3]. Annual mortality rates for these diseases range from 10% to 90% [1,2], even though most patients receive antifungal drug therapy. First-line antifungals, azoles such as fluconazole that target ergosterol biosynthesis, polyenes such as amphotericin B (AmB) that target ergosterol directly, and echinocandins such as anidulafungin that target β-1,3 glucan synthesis, are sometimes ineffective at clearing fungal infections so as to prevent reemergence of the infection [4,5]. Unsatisfactory drug efficacy is also atttributed to other factors such as drug toxicity with extended treatment and the emergence of resistance to the current antifungals [6–8]. To improve antifungal therapy, one critical and challenging approach is to develop new antifungals with novel modes of action. Another approach is to increase the efficacy and reduce toxicity of currently available and future antifungals. At present, antifungals delivered to patients have no particular affinity for fungal cells. Hence, off target effects and host toxicities have significantly limited the efficacy of antifungal therapy. Our goal with DectiSomes is to dramatically improve the effectiveness of antifungal drugs by targeting them to fungal cells and away from host cells (**Fig 1**).

## What are DectiSomes?

DectiSomes represent a novel pan-antifungal drug targeting technology. Current antifungal therapies rely on free antifungal drugs or untargeted liposomal or micellar drugs, which distribute randomly to fungal and mammalian cells alike. By contrast, DectiSomes are drug-loaded liposomes coated with a protein(s) that targets them to fungal cell walls and their exopolysaccharide matrices (**Fig 1A**) [9,10]. DectiSomes bring antifungal drugs in close proximity to fungal cells to increase their local concentrations and to lower their concentration in host cells, thereby lowering the mg/kg effective dose of drug needed to control the pathogen [9–11], reducing host toxicity, and allowing temporally extended treatment regimens. All these effects should lead to better fungal clearance and better patient outcomes.

We’ve made 2 types of DectiSomes so far (**Fig 1B**), based on Dectin-1 [10–12] and Dectin-2 [9], the mammalian immune receptors for oligoglucans and oligomannans, respectively. We first prepared AmB-loaded liposomes, AmB-LLs [10]. AmB-LLs are pegylated analogs of the widely used commercial drug AmBisome. We then coated the AmB-LLs with the carbohydrate recognition domains of Dectin-1 [10,12] and Dectin-2 [9] to make DEC1-AmB-LLs and DEC2-AmB-LLs, respectively. Two Dectin monomers float together and form dimers that bind specifically to oligoglucans and oligomannans found in the cell walls, exopolysaccharide matrices, and biofilms of fungal pathogens [13]. The presence of a large number of Dectin molecules on each liposome ensures efficient and high avidity binding when liposomes encounter fungal ligands at multiple sites on fungal cells. Together, Dectin-1 and Dectin-2 recognize essentially all of the 20 phyla of evolutionarily divergent fungal pathogens including the 5 major pathogens mentioned above [13]. Given that most antifungal drugs are hydrophobic or amphiphobic and may be incorporated efficiently into a liposomal membrane to improve drug tissue penetration and extend their half-life, we anticipated that DectiSomes would function as effective pan-antifungal agents.

## What is the evidence that DectiSomes are more effective than untargeted drugs?

A strong indication that DectiSomes are more effective than untargeted drugs came first from in vitro fungal cell binding and killing studies (**Table 1**). DEC1-AmB-LLs and DEC2-AmB-LLs bind to the cell walls and more efficiently to the exopolysaccharide matrices of in vitro grown *Aspergillus fumigatus*, *Candida albicans*, and *Cryptococcus neoformans* 50- to 200-fold more strongly than untargeted AmB-LLs [9,10]. Binding is oligoglycan cognate specific, such that the oligoglucan laminarin specifically inhibits DEC1-AmB-LL binding [10], and yeast oligomannan specifically inhibits DEC2-AmB-LL binding [10]. DEC1-AmB-LLs kill and/or inhibit the growth of *A*. *fumigatus* cells 5- to 50-fold more efficiently than untargeted AmB-LLs delivering the same concentrations of AmB based on a reduction in rates of conidial germination, a reduction in hyphal extension from germlings, and a loss of metabolic activity of growing hyphae [10]. Similarly, DEC2-AmB-LLs kill and/or inhibit the growth of *A*. *fumigatus*, *C*. *albicans*, and *C*. *neoformans* 2- to 90-fold more efficiently than untargeted AmB-LLs using a range of AmB concentrations [9].

**Table 1:** Performance of Dectin-coated AmB-loaded liposomes targeted to fungal cells, relative to untargeted AmB-LLs.

**A. In vitro assays**						
**DectiSomes**	**Pathogen (*disease*)**		**Increase in liposome binding**^**a**^		**Increase in killing/growth inhibition**^**a**^	
DEC1-AmB-LLs	*A*. *fumigatus* (aspergillosis)		>200-fold		5- to 50-fold	
	*C*. *albicans* (candidiasis)		Yes^b^		N.D.^c^	
	*C*. *neoformans* (cryptococcosis)		Yes^b^		N.D.^c^	
DEC2-AmB-LLs	*A*. *fumigatus* (aspergillosis)		>50-fold		>30-fold	
	*C*. *albicans* (candidiasis)		>100-fold		Approximately 90-fold	
	*C*. *neoformans* (cryptococcosis)		>100-fold		Approximately 10-fold	
**B. In vivo assays in murine models of pulmonary aspergillosis**						
**DEC2-AmB-LLs**	**Increase in liposome binding in lungs**^**a**^	**Reduction in fungal burden in the lungs**^**a**^		**Increase in days of survival vs AmB-LLs**^**d**^		**Increase in percent of surviving mice**^**d**^
Neutropenic model	30-fold	12- to 42-fold		3.5 to 18.4 days		8% to 58%
Steroid model	N.D.^c^	8- to 22-fold		N.D.^c^		N.D.^c^

^a^Relative to untargeted AmB-LL delivering the same AmB concentration.

^b^Qualitative observation.

^c^N.D., not done.

^d^Survival study terminated 24 days after infection.

Another indication that DectiSomes are more effective than untargeted drugs came from in vivo studies using animal models of mycoses (**Table 1B**). We employed a neutropenic/leukopenic mouse model of pulmonary aspergillosis [11], which mimics the chemo-immunosuppression of patients with solid tumors, hematologic malignancies, or immune disorders such as AIDS and patients preconditioned for stem cell mobilization and hematopoietic cell transplantation. DEC2-AmB-LLs delivered by oropharyngeal aspiration bind 30-fold more efficiently to exopolysaccharide associated with fungal infection sites in the lungs than AmB-LLs [11]. DEC2-AmB-LL treatments delivering AmB at 0.2 mg/kg dose reduce the fungal cell burden in the lungs 12- to 42-fold more efficiently than AmB-LLs [11]. This is a 25-fold lower dose than that reported for the AmB-LL analog AmBisome (5 mg/kg) to produce an order of magnitude reduction in fungal burden in related mouse models or recommended for clinical treatment of aspergillosis [11]. At 0.2 mg/kg AmB, DEC2-AmB-LLs dramatically improve mouse survival relative to AmB-LLs [11]. We observed a similar drop in lung fungal burden in a steroid immunosuppression mouse model of pulmonary aspergillosis using a similar treatment regimen [11]. This latter murine model mimics the glucocorticoid immunosuppression of patients for diseases of chronic inflammation such as asthma and rheumatoid arthritis, for some leukemias, and for post-organ transplantation therapy. We are currently examining the efficacy of DectiSomes in mouse models of invasive candidiasis, pulmonary mucormycosis, and cryptococcal meningitis.

In summary, both in vitro and in vivo data show that Dectin targeting of liposomal AmB dramatically improves drug efficacy and lowers the effective dose. Yet there are concerns, particularly with species such as *C*. *neoformans*, which is coated with a thick capsule composed of biochemically complex exopolysaccharides and the capsule sheds copiously during infection. DectiSomes bound to soluble fungal glycans might have reduced antifungal activity. Furthermore, liposomes are only moderately effective at penetrating the blood–brain barrier. We do not know if coating them with dectins will help or hinder this limitation. We hope to address these issues experimentally in the near future.

## Why use dectins and not monoclonal antibodies in designing targeted liposomes?

DectiSomes are in essence a novel type of immunoliposomes. Traditionally, immunoliposomes are liposomes targeted to disease cells by monoclonal antibodies. These antibodies enable the delivery of bundled anticancer drugs specifically to tumors or dispersed cancer cells and away from normal host cells. Antibody-targeted immunoliposomes generally increase cancer cell-type specificity and reduce host cytotoxicity by 5- to 10-fold over passive delivery of anticancer drugs [14,15]. Several antibody-targeted immunoliposomal pharmaceuticals have been FDA approved. So why not use traditional immunoliposomes to improve the performance of antifungal drugs?

We were concerned that antibodies might not be as efficacious an approach for targeting liposomes to fungal pathogens for 2 primary reasons. First, high-affinity monoclonal antibodies made to fungal cell antigens may be too specific to allow development of a pan-antifungal drug delivery system [16]. They may react with only a few genera of fungi [16] or only recognize a specific growth stage of a single species [17]. Nor is it clear if any single monoclonal could recognize the numerous different variants in glycan structures. That said, an antibody may be identified in the future that is suitable for targeting drug-loaded liposomes. By contrast, Dectin-1 and Dectin-2 recognize most fungal pathogens [13] and have been shown to recognize different stages of cell development [18,19]. Respectively, they recognized diverse crosslink variants of beta-glucans [20] and alpha-mannans [21,22]. The avidity created by having more than 1,000 Dectin molecules concentrated on each liposome [9,10] should compensate for low-affinity epitopes. Secondly, the high cost of producing monoclonal antibodies is inhibitory [23]. This is a serious concern given that many of the patients of invasive mycoses are from resource-limited regions. We developed methods of biochemically manipulating and producing functional carbohydrate recognition domains of dectins in *Escherichia coli* at a few percent of the cost of producing and manipulating the same molar concentrations of monoclonal antibodies in cultured mammalian cells [9,10]. Low reagent protein cost and high-affinity binding to diverse pan-fungal pathogens are essential for DectiSomes to have a global impact on fungal disease thearapy.

## What needs to be done to justify clinical trials on DectiSomes and ensure sufficient financial investment?

Even with strong governmental support for the clinical development of new antifungal drugs, the process for just one new drug takes a major commitment of drug industry resources and an investment of 200 to 300 million US dollars [6]. Thus, much remains to be done to make DectiSomes a clinical reality. (**i**) Showing the efficacy of DectiSomes in mouse models of more and diverse fungal diseases beyond aspergillosis, including candidiasis, cryptococcosis, mucormycosis, and PCP, will establish the pan-antifungal properties of this technology and give drug companies the rationale and incentive to invest in the technology. (**ii**) Demonstrating efficacy against systemic as well as other types of fungal infections such as fungal keratitis and onychomycosis would dramatically expand the potential patient populations and market for DectiSome-based antifungals. (**iii**) Showing that DectiSome targeting improves the performance of azole and echinocandin antifungals would allow drug companies to repurpose all 3 major classes of antifungal drugs currently off patent. We are particularly interested in testing if DectiSomes loaded with echinocandins could be used to treat cryptococcosis. Currently, echinocandins are not effective against *Cryptococcus* either in vitro or in vivo, despite the fact that cryptococcal β-1,3-glucan synthase is highly sensitive to echinocadins [24]. (**iv**) Demonstrating that increased drug efficacy of DectiSomes can overcome some forms of dose-dependent resistance for existing drugs will further expand their clinical use. (**v**) The human dectins share the same predicted 3D structures as their mouse paralogs, but are 30% to 40% divergent in their amino acids sequences. Parallel studies are needed on the human dectins. (**vi**) Finally, establishing the efficacy of the targeted delivery of liposomal drugs to the extracellular oligoglycans of protozoan parasites would open an enormous new field of application [25].

## References

[1] Prakash H, Chakrabarti A. Global Epidemiology of Mucormycosis. J Fungi (Basel). 2019;5(1). Epub 2019/03/25. doi: 10.3390/jof5010026 ; PubMed Central PMCID: PMC6462913. Available from: https://www.ncbi.nlm.nih.gov/pubmed/30901907 30901907 PMC6462913

[2] Bongomin F, Gago S, Oladele R, Denning D. Global and Multi-National Prevalence of Fungal Diseases-Estimate Precision. J Fungi (Basel). 2017;3(4):1–29. Epub 2018/01/27. doi: 10.3390/jof3040057 ; PubMed Central PMCID: PMC5753159. Available from: https://www.ncbi.nlm.nih.gov/pubmed/29371573 29371573 PMC5753159

[3] Benedict K, Jackson B, Chiller T, Beer K. Estimation of Direct Healthcare Costs of Fungal Diseases in the United States. Clin Infect Dis. 2019;68(11):1791–7. Epub 2018/09/12. doi: 10.1093/cid/ciy776 ; PubMed Central PMCID: PMC6409199. Available from: https://www.ncbi.nlm.nih.gov/pubmed/30204844 30204844 PMC6409199

[4] Armstrong-James D, Meintjes G, Brown G. A neglected epidemic: fungal infections in HIV/AIDS. Trends Microbiol. 2014;22(3):120–7. 10.1016/j.tim.2014.01.001. Available from: https://www.sciencedirect.com/science/article/pii/S0966842X1400002X 24530175

[5] O’Connor L, Van Anh D, Chau T, Chau N, Huong L, Wolbers M, et al. Antifungal susceptibility does not correlate with fungal clearance or survival in AIDS-associated cryptococcal meningitis. Clin Infect Dis. 2020. Epub 2020/10/15. doi: 10.1093/cid/ciaa1544 . Available from: https://www.ncbi.nlm.nih.gov/pubmed/33051650 33051650 PMC8561241

[6] Tillotson J, Tillotson G. The Regulatory Pathway for Antifungal Drugs: A US Perspective. Clin Infect Dis. 2015;61(Suppl_6):S678–S83. doi: 10.1093/cid/civ819 Available from: 10.1093/cid/civ819 26567287

[7] Nicola A, Albuquerque P, Paes H, Fernandes L, Costa F, Kioshima E, et al. Antifungal drugs: New insights in research & development. Pharmacol Ther. 2019;195:21–38. Epub 2018/10/23. doi: 10.1016/j.pharmthera.2018.10.008 . Available from: https://www.ncbi.nlm.nih.gov/pubmed/30347212 30347212

[8] Su H, Han L, Huang X. Potential targets for the development of new antifungal drugs. J Antibiot. 2018;71(12):978–91. Epub 2018/09/23. doi: 10.1038/s41429-018-0100-9 . Available from: https://www.ncbi.nlm.nih.gov/pubmed/30242283 30242283

[9] Ambati S, Ellis E, Lin J, Lin X, Lewis A, Meagher R. Dectin-2-Targeted Antifungal Liposomes Exhibit Enhanced Efficacy. mSphere. 2019;4(5):1–16. doi: 10.1128/mSphere.00715-19 . Available from: https://www.ncbi.nlm.nih.gov/pubmed/31666315 31666315 PMC6821932

[10] Ambati S, Ferarro A, Kang S, Lin J, Lin X, Momany M, et al. Dectin-1-Targeted Antifungal Liposomes Exhibit Enhanced Efficacy. mSphere. 2019;4:1–15. Epub 2019/02/15. doi: 10.1128/mSphere.00025-19 ; PubMed Central PMCID: PMC6374590. Available from: https://www.ncbi.nlm.nih.gov/pubmed/30760610 30760610 PMC6374590

[11] Ambati S, Ellis E, Pham T, Lewis Z, Lin X, Meagher R. Antifungal Liposomes Directed by Dectin-2 Offer a Promising Therapeutic Option for Pulmonary Aspergillosis. mBio. 2021;12(1):1. 10.1128/mBio.00030-21 PMC8545082 33622715

[12] Meagher R, Lewis Z, Lin X, Momany M, inventors; University of Georgia Research Foundation, Inc. (Athens, GA, US), assignee. Targeted Nanoparticles and Their Uses Related to Fungal Infections. United States. 2019. Available from: https://patentscope.wipo.int/search/en/detail.jsf?docId=WO2020146514&_cid=P11-KHDI99-36314-1

[13] Goyal S, Castrillon-Betancur J, Klaile E, Slevogt H. The Interaction of Human Pathogenic Fungi With C-Type Lectin Receptors. Front Immunol. 2018;9:1261–85. Epub 2018/06/20. doi: 10.3389/fimmu.2018.01261 ; PubMed Central PMCID: PMC5994417. Available from: https://www.ncbi.nlm.nih.gov/pubmed/29915598 29915598 PMC5994417

[14] Merino M, Zalba S, Garrido M. Immunoliposomes in clinical oncology: State of the art and future perspectives. J Control Release. 2018;275:162–76. Epub 2018/02/16. doi: 10.1016/j.jconrel.2018.02.015 . Available from: https://www.ncbi.nlm.nih.gov/pubmed/29448116 29448116

[15] Eloy J, Petrilli R, Trevizan L, Chorilli M. Immunoliposomes: A review on functionalization strategies and targets for drug delivery. Colloids Surf B Biointerfaces. 2017;159:454–67. doi: 10.1016/j.colsurfb.2017.07.085 . Available from: https://www.ncbi.nlm.nih.gov/pubmed/28837895 28837895

[16] Lion T. Human Fungal Pathogen Identification: Method and Protocols. 3.2 Galactomannan (GM) Immunoassay for Acute Aspergillosis. Vienna, Austria: Humana Press; 2017. p. 439.

[17] Momany M, Lindsey R, Hill T, Richardson E, Momany C, Pedreira M, et al. The *Aspergillus fumigatus* cell wall is organized in domains that are remodelled during polarity establishment. Microbiology. 2004;150(Pt 10):3261–8. Epub 2004/10/08. doi: 10.1099/mic.0.27318-0 . Available from: https://www.ncbi.nlm.nih.gov/pubmed/15470106 15470106

[18] Gersuk G, Underhill D, Zhu L, Marr K. Dectin-1 and TLRs permit macrophages to distinguish between different *Aspergillus fumigatus* cellular states. J Immunol. 2006;176(6):3717–24. Epub 2006/03/07. doi: 10.4049/jimmunol.176.6.3717 . Available from: https://www.ncbi.nlm.nih.gov/pubmed/16517740 16517740

[19] Steele C, Rapaka R, Metz A, Pop S, Williams D, Gordon S, et al. The beta-glucan receptor dectin-1 recognizes specific morphologies of *Aspergillus fumigatus*. PLoS Pathog. 2005;1(4):e42. Epub 2005/12/14. doi: 10.1371/journal.ppat.0010042 ; PubMed Central PMCID: PMC1311140. Available from: https://www.ncbi.nlm.nih.gov/pubmed/16344862 16344862 PMC1311140

[20] Adams E, Rice P, Graves B, Ensley H, Yu H, Brown G, et al. Differential high-affinity interaction of dectin-1 with natural or synthetic glucans is dependent upon primary structure and is influenced by polymer chain length and side-chain branching. J Pharmacol Exp Ther. 2008;325(1):115–23. Epub 2008/01/04. doi: 10.1124/jpet.107.133124 . Available from: https://www.ncbi.nlm.nih.gov/pubmed/18171906 18171906

[21] Feinberg H, Jegouzo S, Rex M, Drickamer K, Weis W, Taylor M. Mechanism of pathogen recognition by human Dectin-2. J Biol Chem. 2017;292(32):13402–14. Epub 2017/06/28. doi: 10.1074/jbc.M117.799080 ; PubMed Central PMCID: PMC5555199. Available from: https://www.ncbi.nlm.nih.gov/pubmed/28652405 28652405 PMC5555199

[22] McGreal P, Rosas M, Brown G, Zamze S, Wong S, Gordon S, et al. The carbohydrate-recognition domain of Dectin-2 is a C-type lectin with specificity for high mannose. Glycobiology. 2006;16(5):422–30. Epub 2006/01/21. doi: 10.1093/glycob/cwj077 . Available from: https://www.ncbi.nlm.nih.gov/pubmed/16423983 16423983

[23] Hernandez I, Bott S, Patel A, Wolf C, Hospodar A, Sampathkumar S, et al. Pricing of monoclonal antibody therapies: higher if used for cancer? Am J Manag Care. 2018;24(2):109–12. Epub 2018/02/21. . 29461857

[24] Maligie M, Selitrennikoff C. Cryptococcus neoformans resistance to echinocandins: (1,3)beta-glucan synthase activity is sensitive to echinocandins. Antimicrob Agents Chemother. 2005;49(7):2851–6. doi: 10.1128/AAC.49.7.2851-2856.2005 . Available from: https://pubmed.ncbi.nlm.nih.gov/15980360 https://www.ncbi.nlm.nih.gov/pmc/articles/PMC1168702/15980360 PMC1168702

[25] Cummings R, van Die I. Parasitic Infections. In: Varki A, Cummings R, Esko J, editors. Essentials of Glycobiology. 3rd ed. Cold Spring Harbor, NY: Cold Spring Harbor Laboratory Press; 2017. Available from: https://www.ncbi.nlm.nih.gov/books/NBK453068/

